# Diagnostic value of multiparametric MRI radiomics combined with serum PSA and tissue P504S for prostate cancer

**DOI:** 10.1097/MD.0000000000051391

**Published:** 2026-11-06

**Authors:** Lu Zhang, Deyuan Zhao, Yujing Bai, Dengcui Zhao, Jian Li

**Affiliations:** a The first hospital of Hebei Medical University, Shijiazhuang, Hebei Province, China.

**Keywords:** Diagnostic Model, Multiparametric MRI, P504S, Prostate Cancer, PSA, Radiomics

## Abstract

This study aimed to evaluate the diagnostic value of multiparametric MRI radiomics features combined with serum PSA-related indicators and tissue P504S expression for prostate cancer, and to develop a multimodal auxiliary diagnostic model in a retrospective cohort that was not restricted to early-stage disease. Clinical, imaging, and pathological data from 126 patients with suspected prostate cancer were retrospectively collected and divided into a prostate cancer group (69 cases) and a benign lesion group (57 cases). All patients underwent prostate MRI; radiomics features were extracted, and LASSO regression, with 10-fold cross-validation for tuning, was used to construct a Rad-score. Serum t-PSA, f-PSA, f/t-PSA ratio, and PSAD were measured. Tissue P504S expression was detected by immunohistochemistry and semi-quantitatively scored. A combined diagnostic model was constructed using multivariate logistic regression. ROC metrics were evaluated in the model-development cohort and are reported as apparent performance. AUC differences between models were compared using the DeLong test. The prostate cancer group had higher t-PSA and PSAD and a lower f/t-PSA ratio than the benign lesion group (all *P* < .001). P504S was positive in 64/69 cancer cases (92.8%) and 9/57 benign cases (15.8%; *P* < .001). Eight radiomics features were selected, and the median Rad-score was 0.86 in cancer cases and − 0.52 in benign cases (*P* < .001). The full model combining Rad-score, f/t-PSA, PSAD, and tissue P504S semiquantitative score achieved an apparent AUC of 0.956 (95% CI: 0.918–0.994), sensitivity of 92.8%, specificity of 91.2%, PPV of 92.8%, and NPV of 91.2%. The overall imaging + serum model had an apparent AUC of 0.912 (95% CI: 0.856–0.968). In exploratory location-based analyses, AUCs were 0.962 for peripheral zone lesions and 0.934 for transition zone lesions. The full model showed promising apparent discrimination for prostate cancer in this retrospective cohort and may represent a potential auxiliary diagnostic model after tissue sampling. The imaging + serum model could potentially support biopsy decision-making, whereas the full model requires tissue P504S assessment and may assist diagnostic confirmation or risk characterization. Neither incremental clinical benefit nor a reduction in unnecessary biopsies has been established. Internal and external validation and evaluation of the practical clinical workflow are required before routine use.

## 1. Introduction

Prostate cancer is one of the most common malignancies of the male genitourinary system, with its incidence and mortality rates continuously rising worldwide.^[1]^ In China, the disease burden of prostate cancer is increasing due to population aging. In 2022, there were 134,000 new cases of prostate cancer in China, ranking sixth in incidence among male malignancies, with an annual growth rate of approximately 7%.^[2]^ More notably, over two-thirds of Chinese prostate cancer patients are diagnosed at intermediate or advanced stages, far exceeding the rate of <10% in European and American countries, resulting in a 5-year survival rate of only 69.2% compared to 99.5% in the United States over the same period.^[2]^ The core reason for this disparity is the lack of specific clinical symptoms in early-stage prostate cancer, causing most patients to miss the optimal treatment window.^[1]^ Therefore, improving the diagnostic assessment of suspected prostate cancer holds significant clinical importance.

Serum prostate-specific antigen (PSA) is the most widely used screening marker for prostate cancer, but its specificity and sensitivity have notable limitations.^[3]^ Particularly within the PSA gray zone (4–10 ng/mL), nonmalignant conditions such as benign prostatic hyperplasia and prostatitis can also elevate PSA levels, leading to numerous unnecessary biopsies.^[4]^ Although derived indicators such as the f/t-PSA ratio and PSA density (PSAD) can improve diagnostic performance to some extent, single serological markers remain insufficient for precise diagnosis.^[5]^ In recent years, multiparametric MRI, with its excellent soft tissue resolution, has become the cornerstone of imaging diagnosis for prostate cancer, and the widespread application of the PI-RADS score has significantly enhanced the standardization of imaging reports.^[6]^ However, for indeterminate lesions with PI-RADS 3 and certain small lesions located in the transition zone, relying solely on imaging assessment still carries a risk of misdiagnosis.^[7]^

P504S (α-methylacyl-CoA racemase) is an ancillary immunohistochemical marker used in prostate pathology. It is frequently expressed in prostate cancer, although positive staining may also occur in benign lesions.^[8]^ Its tissue-based assessment requires biopsy or surgical specimens and therefore becomes available only after tissue sampling. Meanwhile, radiomics, as an emerging quantitative imaging analysis technique, can quantify tumor heterogeneity by high-throughput extraction of features imperceptible to the human eye from medical images, thereby providing a new dimension for prostate cancer diagnosis.^[9]^ Theoretically, MRI radiomics provides information on tumor heterogeneity, PSA reflects prostate secretory function, and P504S contributes tissue-level molecular information. These imaging, serum, and pathology dimensions may offer complementary diagnostic information. However, their availability at different points in the diagnostic pathway must be distinguished: an imaging + serum model may be evaluated before biopsy, whereas a full model incorporating tissue P504S can only be applied after tissue sampling.

Based on the above background, this study retrospectively collected clinical data from patients with suspected prostate cancer to assess the diagnostic value of multiparametric MRI radiomics features based on the MDME sequence combined with serum PSA-related indicators and tissue P504S expression. The objectives were to develop multimodal diagnostic models and describe their apparent performance in the development cohort, with exploratory subgroup analyses by Gleason score and lesion location. The imaging + serum model was considered a candidate for assessment before biopsy, while the full imaging + serum + pathology model was considered a potential auxiliary model after tissue sampling. The cohort was not restricted to early-stage prostate cancer, and this study does not establish screening effectiveness or clinical benefit.

## 2. Methods

### 2.1. Study design

This study was approved by the Ethics Committee of The first hospital of Hebei Medical University. This study was a retrospective study. A total of 126 male patients with suspected prostate cancer who met the inclusion and exclusion criteria were retrospectively collected from June 2024 to January 2026 at our hospital. The cohort was not restricted to early-stage disease and included patients with T3 and T4 prostate cancer. Accordingly, the study addresses diagnosis across the pathological spectrum represented in this cohort, rather than diagnosis exclusively in an early-stage population. This was a model-development study; the reported diagnostic performance represents apparent performance in the development cohort.

## 3. Inclusion and exclusion criteria

### 3.1. Inclusion criteria

Patients underwent preoperative multiparametric MRI (MDME sequence); had complete serum PSA test results; had prostate biopsy or surgical pathology results; pathological specimens contained P504S immunohistochemistry results (positive/negative, semiquantitative score); had complete clinical, imaging, and pathological data.

### 3.2. Exclusion criteria

Poor MRI image quality (motion artifacts/metal artifacts) affecting interpretation; contraindications to MRI (e.g., cardiac pacemaker, claustrophobia, etc.); pathological specimens without available P504S results; prior prostate cancer-related treatment (surgery, radiotherapy, endocrine therapy, etc.).

## 4. Detection of key indicators

### 4.1. MRI examination and image post-processing

In this study, a Siemens MAGNETOM Vida 3.0T superconducting MRI scanner and an abdominal 18-channel coil were used for image acquisition. All enrolled patients underwent prostate MDME sequence scanning with the following parameters: TR 4350 ms, TE1 23 ms, TE2 101 ms, FOV 250 × 250 mm, flip angle 150°, slice thickness 4.0 mm. Patients were placed in the supine position with arms naturally relaxed and breathing calmly. After scanning, the original images were uploaded to the syngo via post-processing workstation, and T1-weighted images, T2-weighted images, and proton density-weighted images were generated using MDME software. The largest cross-section of the prostate and the adjacent sections above and below it were selected. Two imaging technicians with 17 years of working experience independently and manually delineated the prostate and lesion boundaries using a double-blind method, avoiding interference from surrounding tissues, and extracted radiomics features from the region of interest, including shape features, first-order statistical features, and texture features.

### 4.2. Serum PSA detection method

Fasting peripheral venous blood (approximately 5 mL) was collected from all patients before biopsy or surgery. After standing to allow clotting, the sample was centrifuged at 3000 r/min for 10 minutes to separate the serum. Serum total PSA (t-PSA) and free PSA (f-PSA) were detected using electrochemiluminescence immunoassay on a Roche Cobas e601 automatic analyzer. The f/t-PSA ratio and PSA density (PSAD) were calculated. Each specimen was tested twice, and the mean value was taken. Internal quality control calibration was completed before testing.

### 4.3. Tissue P504S immunohistochemistry method

Prostate biopsy or surgical resection specimens were fixed in 10% neutral formalin for 6 to 24 hours, routinely dehydrated, paraffin-embedded, and cut into 4 μm sections. Immunohistochemical staining was performed using the EnVision two-step method: sections were dewaxed and rehydrated, followed by citrate buffer high-pressure heat-induced antigen retrieval. Endogenous peroxidase was blocked with 3% hydrogen peroxide. Rabbit anti P504S monoclonal antibody (1:100) was added and incubated overnight at 4°C. The next day, horseradish peroxidase-labeled secondary antibody was added and incubated for 30 minutes at room temperature. DAB color development and hematoxylin counterstaining were performed before mounting. Positive and negative controls were used for quality control. Two pathologists performed double-blind interpretation. Semiquantitative scoring (0–9) was calculated as the product of the percentage of positive cells (0–3) and staining intensity (0–3). A score ≥ 2 was defined as positive. Discrepancies were reviewed by a third senior pathologist.

## 5. General data collection

Clinical, imaging, and pathological data of enrolled patients were retrospectively collected through the hospital’s electronic medical record system and picture archiving and communication system (PACS). Clinical data included demographic characteristics (age, height, weight, BMI), lifestyle habits (smoking history, alcohol consumption history), past medical history (family history of prostate cancer, history of prostatitis), and International Prostate Symptom Score (IPSS). Serological data included serum t-PSA and f-PSA results before biopsy or surgery, and the f/t-PSA ratio and PSAD were calculated. Imaging data included original prostate MDME sequence images, PI-RADS score, total prostate volume, lesion location (peripheral zone/transition zone), and maximum lesion diameter. Pathological data included Gleason score, T stage, and P504S immunohistochemistry results (qualitative and semiquantitative score) after biopsy or surgery. All data were independently cross-checked by two researchers to ensure completeness and accuracy. Discrepancies were resolved through discussion. As P504S was measured in biopsy or surgical tissue, this indicator was available only after tissue sampling. The full model incorporating P504S therefore cannot be used to determine whether an initial tissue-sampling procedure is needed.

## 6. Statistical analysis

Data processing and analysis were performed using SPSS 25.0 and R software (version 4.2). Categorical data were expressed as number (percentage). The categorical comparisons in Tables 1–2 used Pearson χ^2^ tests. Normality of data distribution was assessed using the Kolmogorov-Smirnov test. Normally distributed continuous data were expressed as mean ± standard deviation, and comparisons between groups were made using the independent samples *t*-test. Non-normally distributed continuous data were expressed as median (interquartile range), and comparisons between groups were made using the Mann–Whitney *U* test. Trend analysis of P504S positivity across t-PSA strata used a Cochran–Armitage test with ordered scores 0, 1 and 2. The association between P504S positivity and Gleason score was assessed using a separate Cochran–Armitage trend test. The radiomics feature selection process was as follows: first, the intraclass correlation coefficient (ICC) was calculated to exclude features with poor stability (ICC < 0.75); then, Pearson correlation analysis was used to exclude highly redundant features (*R* > 0.9); finally, LASSO regression, with 10-fold cross-validation for tuning the penalty parameter during feature selection, was used to select the optimal feature subset. The radiomics score (Rad-score) was constructed based on the selected features and their coefficients. Combined diagnostic models were constructed using multivariate logistic regression. Receiver operating characteristic (ROC) curves were plotted to evaluate the apparent diagnostic performance of each indicator and the combined models in the model-development cohort. The area under the curve (AUC), sensitivity, specificity, positive predictive value, negative predictive value, and Youden index were calculated. The diagnostic threshold for each indicator was determined using the maximum Youden index in the development data; threshold-dependent metrics therefore also represent apparent performance. The 10-fold cross-validation used for LASSO tuning should not be interpreted as internal validation of the complete feature-selection and combined-model workflow. No separately estimated internally or externally validated performance of the complete model is reported. Comparison of AUCs between different models was performed using the DeLong test. The Hosmer-Lemeshow test was used to assess model goodness-of-fit in the development data and does not constitute independent model validation. Subgroup analyses by PSA range, Gleason score, and lesion location were considered exploratory; subgroup AUC differences were interpreted descriptively and were not taken as evidence of improved diagnostic accuracy with increasing tumor grade. All tests were two-sided, and a *P*-value < 0.05 was considered statistically significant. For the subgroup analyses in Table 9, all 57 benign controls were included in each Gleason comparison. Full-model subgroup metrics were calculated using one fixed score and a common probability threshold of 0.50 without subgroup refitting. Confidence intervals were calculated using the empirical DeLong variance and normal approximation, with endpoints bounded by 0 and 1. The PSA gray-zone analysis included 30 cancer cases and 22 benign cases, and the f/t-PSA threshold was selected by maximum Youden.

**Table 1:** Clinical, imaging, and pathological characteristics of the study cohort.

Characteristic	Overall (n = 126)	Prostate cancer (n = 69)	Benign lesions (n = 57)	Statistic	*P* value
Age, years	67.8 ± 7.6	70.2 ± 6.9	65.0 ± 7.4	t = 4.052	<.001
Height, cm	170.5 ± 5.4	170.3 ± 5.6	170.7 ± 5.2	t = −0.408	.684
Weight, kg	68.8 ± 9.4	69.5 ± 9.6	67.9 ± 9.1	t = 0.953	.343
BMI, kg/m^2^	23.7 ± 2.7	23.9 ± 2.8	23.4 ± 2.6	t = 1.032	.304
Smoking history	53 (42.1)	32 (46.4)	21 (36.8)	χ^2^ = 1.164	.281
Alcohol consumption history	44 (34.9)	26 (37.7)	18 (31.6)	χ^2^ = 0.511	.475
Family history of prostate cancer	15 (11.9)	12 (17.4)	3 (5.3)	χ^2^ = 4.378	.036
History of prostatitis	23 (18.3)	10 (14.5)	13 (22.8)	χ^2^ = 1.446	.229
IPSS score	10.0 (5.0, 15.0)	9.0 (5.0, 14.0)	12.0 (7.0, 16.0)	Z = −1.982	.047
t-PSA, ng/mL	8.4 (5.0, 14.0)	12.0 (7.5, 18.2)	5.6 (4.0, 8.5)	Z = −5.763	<.001
f-PSA, ng/mL	1.2 (0.7, 2.0)	1.0 (0.6, 1.5)	1.5 (0.9, 2.5)	Z = −3.458	.001
f/t-PSA ratio	0.14 (0.09, 0.20)	0.10 (0.07, 0.14)	0.21 (0.15, 0.27)	Z = −6.891	<.001
PSAD, ng/mL/cm^3^	0.17 (0.11, 0.29)	0.26 (0.17, 0.37)	0.11 (0.08, 0.17)	Z = −6.204	<.001
Gleason score ≤ 6	–	19 (27.5)	–	–	–
Gleason score 7	–	35 (50.7)	–	–	–
Gleason score ≥ 8	–	15 (21.7)	–	–	–
P504S-positive	73 (57.9)	64 (92.8)	9 (15.8)	χ^2^ = 75.870	<.001
P504S semiquantitative score	4.0 (1.0, 6.0)	6.0 (4.0, 7.0)	1.0 (0.0, 1.0)	Z = −8.567	<.001
T1 stage	–	8 (11.6)	–	–	–
T2 stage	–	35 (50.7)	–	–	–
T3 stage	–	21 (30.4)	–	–	–
T4 stage	–	5 (7.2)	–	–	–
PI-RADS ≤ 3	41 (32.5)	10 (14.5)	31 (54.4)	χ^2^ = 22.630	<.001
PI-RADS ≥ 4	85 (67.5)	59 (85.5)	26 (45.6)	–	–
Prostate volume, cm^3^	49.0 (35.0, 66.0)	45.0 (32.0, 58.0)	56.0 (41.0, 73.0)	Z = −2.645	.008
Peripheral zone lesion	78 (61.9)	49 (71.0)	29 (50.9)	χ^2^ = 5.367	.021
Transition zone lesion	48 (38.1)	20 (29.0)	28 (49.1)	–	–
Maximum lesion diameter, mm	17.5 (12.0, 25.0)	22.0 (15.0, 30.0)	14.0 (10.0, 19.0)	Z = −4.618	<.001

**Table 2:** Association of P504S positivity with clinicopathological characteristics.

A P504S positivity by clinical and pathological strata									
Characteristic	Stratum			n	P504S-positive n (%)	Statistic			P value
Disease group	Prostate cancer			69	64 (92.8)	χ^2^ = 75.870			<.001
	Benign lesions			57	9 (15.8)				
Gleason score	≤6			19	16 (84.2)	Trend χ^2^ = 3.236			.072
	7			35	33 (94.3)				
	≥8			15	15 (100.0)				
t-PSA stratum	<4 ng/mL			18	6 (33.3)	Trend χ^2^ = 11.061			<.001
	4–10 ng/mL			52	26 (50.0)				
	>10 ng/mL			56	41 (73.2)				
PSAD, ng/mL/cm^3^	<0.15			54	19 (35.2)	χ^2^ = 20.072			<.001
	≥0.15			72	54 (75.0)				
Lesion location	Peripheral zone			78	52 (66.7)	χ^2^ = 6.403			.011
	Transition zone			48	21 (43.8)				
PI-RADS	≤3			41	11 (26.8)	χ^2^ = 24.132			<.001
	≥4			85	62 (72.9)				

B: Maximum lesion diameter by P504S status									
P504S status		n	Diameter, mm Median (Q1, Q3)				Statistic	*P* value	
Positive		73	21.5 (15.0, 29.0)				Z = −5.124	<.001	
Negative		53	14.0 (10.0, 19.0)						

**Table 9:** Exploratory subgroup analyses.

A: Full-model score by Gleason group and lesion location								
Analysis	Subgroup	Cancer (n)	Benign (n)	Total (n)	AUC (95% CI)	Sensitivity (%)	Specificity (%)	Youden index
Gleason score	≤6	19	57	76	0.921 (0.858–0.983)	84.2	91.2	0.754
	7	35	57	92	0.966 (0.935–0.997)	94.3	91.2	0.855
	≥8	15	57	72	0.978 (0.951–1.000)	100.0	91.2	0.912
Lesion location	Peripheral zone	49	29	78	0.962 (0.924–1.000)	93.9	93.1	0.870
	Transition zone	20	28	48	0.934 (0.858–1.000)	90.0	89.3	0.793

B: PSA gray-zone comparison								
PSA stratum	Indicator	Cancer (n)	Benign (n)	Total (n)	AUC (95% CI)	Sensitivity (%)	Specificity (%)	Youden index
4–10 ng/mL	Full model	30	22	52	0.912 (0.832–0.992)	86.7	77.3	0.639
	f/t-PSA	30	22	52	0.724 (0.584–0.865)	70.0	63.6	0.336

## 7. Results

### 7.1. Clinical, imaging, and pathological characteristics of the study cohort

A total of 126 patients with suspected prostate cancer were included: 69 (54.8%) had prostate cancer, and 57 (45.2%) had benign lesions (Table 1). Cancer patients were older (70.2 ± 6.9 vs 65.0 ± 7.4 years; *P* < .001) and more frequently reported a family history of prostate cancer (17.4% vs 5.3%; *P* = .036). Median IPSS was lower in the cancer group (9.0 vs 12.0; *P* = .047). Cancer patients had a higher median t-PSA (12.0 vs 5.6 ng/mL) and PSAD (0.26 vs 0.11 ng/mL/cm^3^), and a lower median f/t-PSA ratio (0.10 vs 0.21; all *P* < .001). P504S positivity was 64/69 (92.8%) in cancer cases and 9/57 (15.8%) in benign cases (χ^2^ = 75.870; *P* < .001). PI-RADS ≥ 4 was more frequent in cancer cases (85.5% vs 45.6%; *P* < .001), as were peripheral zone lesions (71.0% vs 50.9%; *P* = .021). Maximum lesion diameter was larger (median 22.0 vs 14.0 mm; *P* < .001), whereas prostate volume was smaller (median 45.0 vs 56.0 cm^3^; *P* = .008). Among the 69 cancer cases, Gleason scores were ≤ 6 in 19 (27.5%), 7 in 35 (50.7%), and ≥ 8 in 15 (21.7%). T stages were T1 in 8, T2 in 35, T3 in 21 and T4 in 5 cases. Thus, 26/69 cancer cases (37.7%) had T3 or T4 disease, and the cohort was not restricted to early-stage prostate cancer.

### 7.2. Association of P504S positivity with clinicopathological characteristics

In the full cohort, P504S positivity was 6/18 (33.3%) at t-PSA < 4 ng/mL, 26/52 (50.0%) at 4–10 ng/mL, and 41/56 (73.2%) at > 10 ng/mL (Cochran–Armitage trend χ^2^ = 11.061; *P* < .001; Table 2). Positivity was higher with PSAD ≥ 0.15 than with PSAD < 0.15 ng/mL/cm^3^ (54/72 [75.0%] vs 19/54 [35.2%]; χ^2^ = 20.072; *P* < .001). It was also higher for peripheral zone than transition zone lesions (52/78 [66.7%] vs 21/48 [43.8%]; χ^2^ = 6.403; *P* = .011), and for PI-RADS ≥ 4 than PI-RADS ≤ 3 (62/85 [72.9%] vs 11/41 [26.8%]; χ^2^ = 24.132; *P* < .001). Maximum lesion diameter was larger in P504S-positive patients (median 21.5 mm [Q1–Q3, 15.0–29.0]) than in P504S-negative patients (14.0 mm [10.0–19.0]; Z =−5.124; *P* < .001). Among cancer cases, P504S positivity was 16/19 (84.2%) in patients with Gleason score ≤ 6, 33/35 (94.3%) with score 7, and 15/15 (100.0%) with score ≥ 8, totaling 64 positive cancer cases. The Cochran–Armitage trend test yielded χ^2^ = 3.236 (*P* = .072).

### 7.3. Radiomics feature selection and rad-score construction

A total of 1245 radiomics features were extracted from MDME-derived images. Stability screening retained 892 features, correlation screening retained 206, and LASSO selected eight features with nonzero coefficients (Table 3). Ten-fold cross-validation was used to select the LASSO penalty parameter; it was not an internal validation of the complete combined-model workflow. The selected feature names and coefficients are listed in Table 4. The coefficient for wavelet-HLL_glcm_ClusterShade was 0.352 and that for original_shape_Sphericity was −0.095. These coefficients describe contributions to the fitted score and do not establish specific biological or histopathological mechanisms. Median Rad-score was 0.86 (Q1–Q3, 0.42–1.35) in cancer cases and − 0.52 (−1.00 to −0.18) in benign cases (Z =−7.286; *P* < .001; Table 5).

**Table 3:** Radiomics feature selection process.

Selection step	Criterion	Features retained, n
Initial extraction	All extracted features	1245
Stability screening	ICC ≥ 0.75	892
Correlation screening	Reported correlation criterion: *R* ≤ 0.90	206
LASSO selection	10-fold cross-validation to select λ.min	8

**Table 4:** Selected radiomics features, coefficients, and feature descriptions.

No.	Feature name	Coefficient	Feature description
1	wavelet-HLL_glcm_ClusterShade	0.352	Asymmetry of co-occurring gray-level values
2	original_shape_Maximum2DDiameterColumn	0.287	Maximum in-plane diameter in the row-slice plane
3	log-sigma-2–0_mm_3D_glcm_Correlation	0.245	Linear dependency between neighboring gray levels
4	original_firstorder_Kurtosis	0.218	Fourth standardized moment of the ROI intensity distribution
5	wavelet-HLL_glszm_LargeAreaHighGrayLevelEmphasis	0.196	Joint distribution of large zones and high gray-level values
6	original_glrlm_LongRunHighGrayLevelEmphasis	0.163	Joint distribution of long runs and high gray-level values
7	log-sigma-3–0_mm_3D_ngtdm_Busyness	0.128	Local gray-level change relative to neighboring intensities
8	original_shape_Sphericity	−0.095	Roundness of the segmented ROI relative to a sphere

Rad-score = 0.352 × X_1_ + 0.287 × X_2_ + 0.245 × X_3_ + 0.218 × X_4_ + 0.196 × X_5_ + 0.163 × X_6_ + 0.128 × X_7_ − 0.095 × X_8_.

**Table 5:** Rad-score distributions in the prostate cancer and benign lesion groups.

Group	n	Rad-score Median (Q1, Q3)	Z statistic	*P* value
Prostate cancer	69	0.86 (0.42, 1.35)	−7.286	<.001
Benign lesions	57	−0.52 (−1.00, −0.18)		

### 7.4. Apparent diagnostic performance of individual indicators

Table 6 summarizes individual-indicator performance in the development cohort. Among serum indicators, f/t-PSA had the largest observed AUC (0.856; 95% CI: 0.786–0.926), compared with 0.812 for t-PSA, 0.694 for f-PSA, and 0.841 for PSAD. The P504S semiquantitative score had an AUC of 0.901 (95% CI: 0.843–0.959), sensitivity of 88.4%, and specificity of 87.7% at the reported cutoff of 3.5. Binary P504S positivity, defined as a score ≥ 2, had a sensitivity of 92.8%, specificity of 84.2%, and AUC of 0.885 (95% CI: 0.822–0.948). Rad-score had an AUC of 0.863 (95% CI: 0.796–0.930), sensitivity of 84.1%, and specificity of 82.5% at the reported cutoff of 0.28. The P504S semiquantitative score had the largest observed single-indicator AUC; this descriptive ranking does not establish pairwise statistical superiority. Cutoffs selected by maximum Youden in these data and their associated metrics require validation.

**Table 6:** Apparent diagnostic performance of individual indicators in the development cohort.

Indicator	AUC (95% CI)	cutoff	Sensitivity (%)	Specificity (%)	PPV (%)	NPV (%)	Youden index
t-PSA, ng/mL	0.812 (0.734–0.890)	8.5	76.8	78.9	81.5	73.8	0.558
f-PSA, ng/mL	0.694 (0.601–0.787)	1.0	65.2	68.4	71.4	61.9	0.336
f/t-PSA ratio	0.856 (0.786–0.926)	0.12	85.5	77.2	81.9	81.5	0.627
PSAD, ng/mL/cm^3^	0.841 (0.768–0.914)	0.18	79.7	80.7	83.3	76.7	0.604
P504S positivity	0.885 (0.822–0.948)	Score ≥ 2	92.8	84.2	87.7	90.6	0.770
P504S semiquantitative score	0.901 (0.843–0.959)	3.5	88.4	87.7	89.7	86.2	0.761
Rad-score	0.863 (0.796–0.930)	0.28	84.1	82.5	85.3	81.0	0.665

AUC = area under the ROC curve, CI = confidence interval, NPV = negative predictive value, PPV = positive predictive value.

### 7.5. Combined model construction and comparison of apparent diagnostic performance

Four combined models were evaluated in the development cohort (Table 7). Model 1 (imaging + serum) had an apparent AUC of 0.912 (95% CI: 0.856–0.968), sensitivity of 87.0%, and specificity of 86.0%. Model 2 (imaging + pathology) and Model 3 (serum + pathology) had apparent AUCs of 0.935 (95% CI: 0.886–0.984) and 0.928 (95% CI: 0.876–0.980), respectively. Model 4, integrating Rad-score, f/t-PSA, PSAD, and tissue P504S semiquantitative score, had the largest observed AUC (0.956; 95% CI: 0.918–0.994), sensitivity of 92.8%, specificity of 91.2%, PPV of 92.8%, and NPV of 91.2%. The paired DeLong comparisons in Table 8 showed higher AUCs for the full model than for P504S semiquantitative score (difference 0.055; *P* = .012), Rad-score (0.093; *P* < .001), f/t-PSA (0.100; *P* < .001), and Model 1 (0.044; *P* = .016). Differences from Models 2 and 3 did not reach statistical significance (*P* = .252 and *P* = .150), which does not establish equivalence. The imaging + serum model uses information available before tissue sampling, whereas Models 2–4 require tissue P504S. These discrimination comparisons do not establish incremental clinical benefit or a reduction in initial biopsies.

**Table 7:** Apparent diagnostic performance of combined models in the development cohort.

Model	AUC (95% CI)	Sensitivity (%)	Specificity (%)	PPV (%)	NPV (%)
Model 1 Imaging + serum	0.912 (0.856–0.968)	87.0	86.0	88.2	84.5
Model 2 Imaging + pathology	0.935 (0.886–0.984)	89.9	89.5	91.2	87.9
Model 3 Serum + pathology	0.928 (0.876–0.980)	88.4	87.7	89.7	86.2
Model 4 Imaging + serum + pathology	0.956 (0.918–0.994)	92.8	91.2	92.8	91.2

Imaging denotes Rad-score; serum denotes f/t-PSA and PSAD; pathology denotes tissue P504S semiquantitative score. AUC and threshold-dependent metrics are apparent estimates in the model-development cohort. PPV and NPV depend on the cancer prevalence in this selected cohort.

**Table 8:** Pairwise DeLong comparisons of apparent AUCs.

Full model compared with	AUC difference	95% CI for difference	Z statistic	*P* value
P504S semiquantitative score	0.055	0.012 to 0.098	2.521	.012
Rad-score	0.093	0.038 to 0.148	3.312	<.001
f/t-PSA ratio	0.100	0.041 to 0.159	3.328	<.001
Model 1 Imaging + serum	0.044	0.008 to 0.080	2.398	.016
Model 2 Imaging + pathology	0.021	-0.015 to 0.057	1.145	.252
Model 3 Serum + pathology	0.028	-0.010 to 0.066	1.440	.150

AUC difference = AUC of the full model − AUC of the comparator, evaluated in the same development cohort.

### 7.6. Exploratory subgroup analyses

Exploratory subgroup analyses are presented in Table 9. For each Gleason group, all 57 benign controls were included, with total analysis samples of 76, 92 and 72 for scores ≤ 6, 7 and ≥ 8, respectively. One fixed full-model score and a common probability threshold of 0.50 were used without subgroup refitting. AUCs were 0.921 (95% CI: 0.858–0.983), 0.966 (95% CI: 0.935–0.997), and 0.978 (95% CI: 0.951–1.000), respectively; sensitivities were 84.2%, 94.3%, and 100.0%, with specificity of 91.2% in each comparison. The cancer-count-weighted AUC across the three Gleason groups was 0.956. For peripheral zone lesions (49 cancer and 29 benign cases) and transition zone lesions (20 cancer and 28 benign cases), AUCs were 0.962 (95% CI: 0.924–1.000) and 0.934 (95% CI: 0.858–1.000), with sensitivities of 93.9% and 90.0% and specificities of 93.1% and 89.3%, respectively. The 52 PSA gray-zone cases comprised 30 cancer and 22 benign cases. AUCs were 0.912 (95% CI: 0.832–0.992) for the full model and 0.724 (95% CI: 0.584–0.865) for f/t-PSA; corresponding sensitivities/specificities were 86.7%/77.3% and 70.0%/63.6%. Numerical subgroup differences do not establish increasing diagnostic accuracy with tumor grade, stable anatomical performance, or clinical utility.

## 8. Discussion

Through a retrospective analysis of 126 patients with suspected prostate cancer, this study evaluated the diagnostic value of combining multiparametric MRI radiomics, serum PSA-related indicators, and tissue P504S expression.^[10]^ The full combined model achieved an apparent AUC of 0.956, with sensitivity of 92.8% and specificity of 91.2%, and its AUC was higher than that of each single indicator in the development cohort. The imaging + serum model achieved an apparent AUC of 0.912 in the overall cohort. These findings suggest that imaging, serum, and tissue information may provide complementary discrimination, consistent with the rationale for multimodal diagnostic assessment.^[11]^ However, the cohort included T3 and T4 cancers and was not restricted to early-stage disease. The results therefore concern diagnosis in this selected cohort and do not establish early-stage detection, validated model performance, or clinical utility.

First, in this study, the positivity rate of P504S was 92.8% in the prostate cancer group and 15.8% in the benign lesion group, and its semiquantitative score achieved an AUC of 0.901, the highest among the individual indicators. As α-methylacyl-CoA racemase, P504S is associated with altered lipid metabolism in prostate cancer cells.^[12]^ Prior research has examined associations between P504S expression and pathological grade.^[13]^ In this cohort, the grade-stratified positivity trend was not statistically significant (*P* = .072), and these findings do not establish P504S as a prognostic or risk-stratification tool. The semiquantitative cutoff of ≥ 3.5 was selected from these development data and should not be treated as a validated clinical decision threshold. Tissue P504S results become available only after biopsy or surgery and should be interpreted alongside histomorphology and other relevant pathological findings.^[14]^ P504S positivity therefore cannot guide the decision to perform an initial biopsy, and a negative result does not, by itself, justify deferring further evaluation when clinical suspicion persists.

Second, among serological indicators, the f/t-PSA ratio had an AUC of 0.856, outperforming t-PSA and PSAD, which is consistent with clinical knowledge.^[15]^ t-PSA is easily influenced by factors such as prostate volume and inflammation, whereas the f/t-PSA ratio may improve specificity for prostate cancer. In this study, the total prostate volume in the benign lesion group was significantly larger than that in the malignant group (56.0 cm^3^ vs 45.0 cm^3^), which may partly explain false-positive t-PSA findings in patients with benign hyperplasia. In exploratory PSA gray-zone analyses, AUCs were 0.724 for f/t-PSA and 0.912 for the full model (Table 9), suggesting possible complementary information in this diagnostically challenging subgroup.^[16]^ This subgroup estimate for the full model is distinct from the overall-cohort AUC of 0.912 for the imaging + serum model. It suggests possible complementary information in a diagnostically challenging subgroup but does not establish suitability for biopsy decision-making because the full model requires tissue P504S results.

In this study, eight radiomics features were selected from 1245 extracted features, and Rad-score had an apparent AUC of 0.863.^[17]^ The selected descriptors quantify image intensity distributions, spatial texture patterns, and ROI shape. The coefficient for ClusterShade was positive and that for Sphericity was negative, but their signs and magnitudes do not demonstrate cell density, nuclear atypia, or specific molecular mechanisms. Relationships between quantitative imaging and tissue markers require dedicated analyses and validation.^[18]^

After integrating the three categories of indicators, the full combined model achieved an apparent AUC of 0.956. Differences compared with the imaging + pathology model (0.935) and serum + pathology model (0.928) were not statistically significant. This finding does not establish equivalence or identify which component provides the principal diagnostic contribution. Clinically, the models have different potential uses. The imaging + serum model (overall-cohort AUC 0.912) uses information available before tissue sampling and could potentially support biopsy decision-making after appropriate validation. The full imaging + serum + pathology model requires tissue P504S results and may be explored as an auxiliary model for diagnostic confirmation or risk characterization after tissue sampling. Its incremental value over routine histopathological assessment remains unproven, and it cannot be used to avoid the initial tissue-sampling procedure required to obtain P504S. In the high-grade subgroup (Gleason score ≥ 8), the full model had an AUC of 0.978 and sensitivity of 100% (Table 9). These exploratory estimates do not establish increasing diagnostic accuracy with tumor grade or reliable detection of all high-grade cancers. Associations between MRI heterogeneity and Gleason grade described in previous research^[19]^ provide biological context but do not validate the observed between-subgroup differences.

This study has several limitations. First, as a single-center retrospective study with 126 patients, it may be subject to selection bias, and its generalizability requires evaluation in prospective multicenter cohorts. The inclusion of T3 and T4 cancers also limits extrapolation to early-stage disease or population screening. Second, the reported ROC metrics describe apparent performance in the model-development cohort. Cross-validation was used for LASSO tuning during feature selection, but separately estimated internal validation of the complete modeling workflow and external validation are not reported. The apparent AUCs and data-derived thresholds may be optimistic; internal validation should repeat the complete feature-selection and model-fitting process within resampling, followed by evaluation in independent cohorts. Third, subgroup analyses were exploratory, and numerical AUC differences may reflect limited subgroup size, case mix, and sampling variability rather than reproducible differences in accuracy. Fourth, radiomics feature extraction relied on manual ROI delineation. Although performed by two senior technicians using a double-blind method, operator dependence cannot be completely avoided; future studies could evaluate automated segmentation. Fifth, the MDME sequence used in this study did not include DWI/ADC, which may limit the information captured by the radiomics approach. Sixth, P504S detection requires biopsy or surgical specimens, restricting the full model to use after tissue sampling. Potential diagnostic information overlap between tissue P504S and the pathological reference diagnosis should be considered, including possible incorporation bias if P504S contributed to the reference classification. Finally, promising discrimination and a higher AUC do not establish incremental clinical benefit. The full model requires comparison with routine histopathological assessment, and both models require further evaluation of calibration, clinical net benefit, generalizability, reproducibility, and practical workflow, including processing time, cost, and standardization of radiomics and P504S scoring. A reduction in unnecessary biopsies or improved patient outcomes was not demonstrated in this study.

In conclusion, the full model integrating multiparametric MRI radiomics, serum PSA-related indicators, and tissue P504S showed promising apparent discrimination for prostate cancer in this retrospective development cohort. It may represent a potential auxiliary diagnostic model for diagnostic confirmation or risk characterization after tissue sampling. The imaging + serum model, with an overall-cohort apparent AUC of 0.912, could potentially support biopsy decision-making after validation. Because the cohort was not restricted to early-stage disease and the full model requires tissue P504S, these findings do not establish effectiveness for early-stage screening or avoidance of initial biopsy. Exploratory subgroup findings should be interpreted cautiously. Internal and external validation and assessment of incremental clinical benefit, generalizability, and the practical clinical workflow are required before either model can be recommended for routine use.

## Author contributions

**Conceptualization:** Lu Zhang, Deyuan Zhao, Yujing Bai, Dengcui Zhao, Jian Li.

**Data curation:** Lu Zhang, Deyuan Zhao, Yujing Bai, Dengcui Zhao, Jian Li.

**Formal analysis:** Lu Zhang, Deyuan Zhao, Yujing Bai, Dengcui Zhao, Jian Li.

**Funding acquisition:** Jian Li.

**Investigation:** Jian Li.

**Writing – original draft:** Jian Li.

**Writing – review & editing:** Jian Li.
